# Multi-enzyme pyruvate removal system to enhance (*R*)-selective reductive amination of ketones†

**DOI:** 10.1039/d0ra06140a

**Published:** 2020-08-05

**Authors:** Jinhua Zhang, Yanshu Zhao, Chao Li, Hao Song

**Affiliations:** Frontier Science Center for Synthetic Biology, Key Laboratory of Systems Bioengineering (MOE), School of Chemical Engineering and Technology, Tianjin University Tianjin 300350 P. R. China hsong@tju.edu.cn +86-18722024233

## Abstract

Biocatalytic transamination is widely used in industrial production of chiral chemicals. Here, we constructed a novel multi-enzyme system to promote the conversion of the amination reaction. Firstly, we constructed the ArR-ωTA/TdcE/FDH/LDH multi-enzyme system, by combination of (*R*)-selective ω-transaminase derived from *Arthrobacter* sp. (ArR-ωTA), formate dehydrogenase (FDH) derived from *Candida boidinii*, formate acetyltransferase (TdcE) and lactate dehydrogenase (LDH) derived from *E. coli* MG1655. This multi-enzyme system was used to efficiently remove the by-product pyruvate by TdcE and LDH to facilitate the transamination reaction. The TdcE/FDH pathway was found to dominate the by-product pyruvate removal in the transamination reaction. Secondly, we optimized the reaction conditions, including d-alanine, DMSO, and pyridoxal phosphate (PLP) with different concentration of 2-pentanone (as a model substrate). Thirdly, by using the ArR-ωTA/TdcE/FDH/LDH system, the conversions of 2-pentanone, 4-phenyl-2-butanone and cyclohexanone were 84.5%, 98.2% and 79.3%, respectively.

## Abstract 2

The ArR-ωTA/TdcE/FDH/LDH system is an efficient system for increasing the conversion in the transamination reaction.

## Graphical abstract

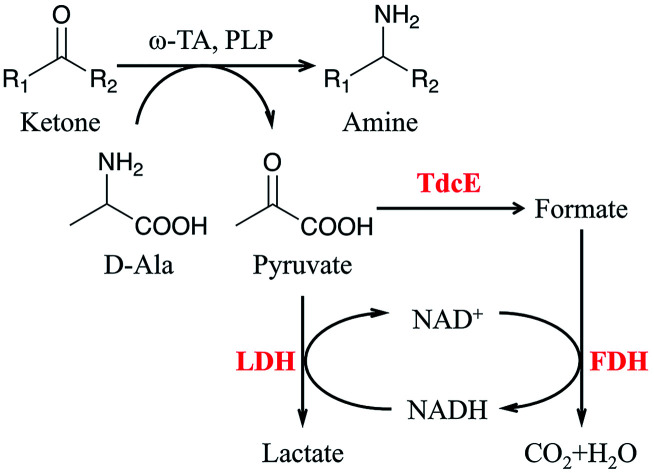

## 1. Introduction

Chiral amines are a class of compounds containing amino groups in the chiral center of small molecule compounds. They are widely used in pharmaceutical and agricultural fields, such as neurological, cardiovascular, antihypertensive, anti-infective drugs, and vaccines, which play an important role in the national economy.^1–5^ At present, the main methods for the preparation of chiral amines^6–9^ are chemical synthesis, biological resolution and biological asymmetric synthesis. Chiral amines synthesized by chemical methods have many shortcomings, such as the need to use expensive metal catalysts, high production costs, low enantioselectivity, and environmental pollution.^10–14^ The maximum theoretical conversion of chiral amine prepared by biological resolution is only 50%.^15^ Thus, chemical methods and biological resolution cannot meet the needs of industrial production. Biological asymmetric synthesis method becomes the preferred strategy for the production of chiral amines because of its theoretical conversion of up to 100%.^16–19^ Therefore, the synthesis of chiral amines by biological asymmetric synthesis has been of increasing interest.

ω-Transaminases (ω-TAs) are pyridoxal phosphate (PLP) dependent enzymes that could be used for the synthesis of chiral amines.^20–23^ Since the transamination reaction catalysed by ω-TA is a reversible reaction, there are two methods to make the reaction proceed in the direction of chiral amines. One of them is employing excess amino donors in a single enzyme catalytic system to drive the transamination reaction and push the reaction toward the synthesis of chiral amines as much as possible;^24^ the other is eliminating the substrate (*i.e.*, by-product) inhibition through a multi-enzyme coupled catalytic reaction, thereby proceeding the reaction toward chiral amine synthesis.^25,26^ Therefore, effective removal of the by-product, pyruvate, is a key factor in the development of an efficient asymmetric amination system.^27–29^ Several approaches to remove pyruvate were reported to improve the conversion of ketones to amines.^29^ Lactate dehydrogenase (LDH) along with ω-TA was used to convert pyruvate to lactate in transamination reactions.^30^ Glucose dehydrogenase (GDH) was introduced in the ω-TA/LDH system to recycle the cofactor NADH.^31^ Thus, the ω-TA/LDH/GDH system has been used for conventional asymmetric reductive amination and synthesized a series of valuable amines.^31–33^ However, the conversion rate of asymmetric amines obtained with the (*R*)-selective ω-transaminase derived from *Arthrobacter* sp. (ArR-ωTA)/LDH/GDH system remained low. Therefore, the conversion rate of amines from the corresponding ketones needs further improvement.

**Fig. 1:**
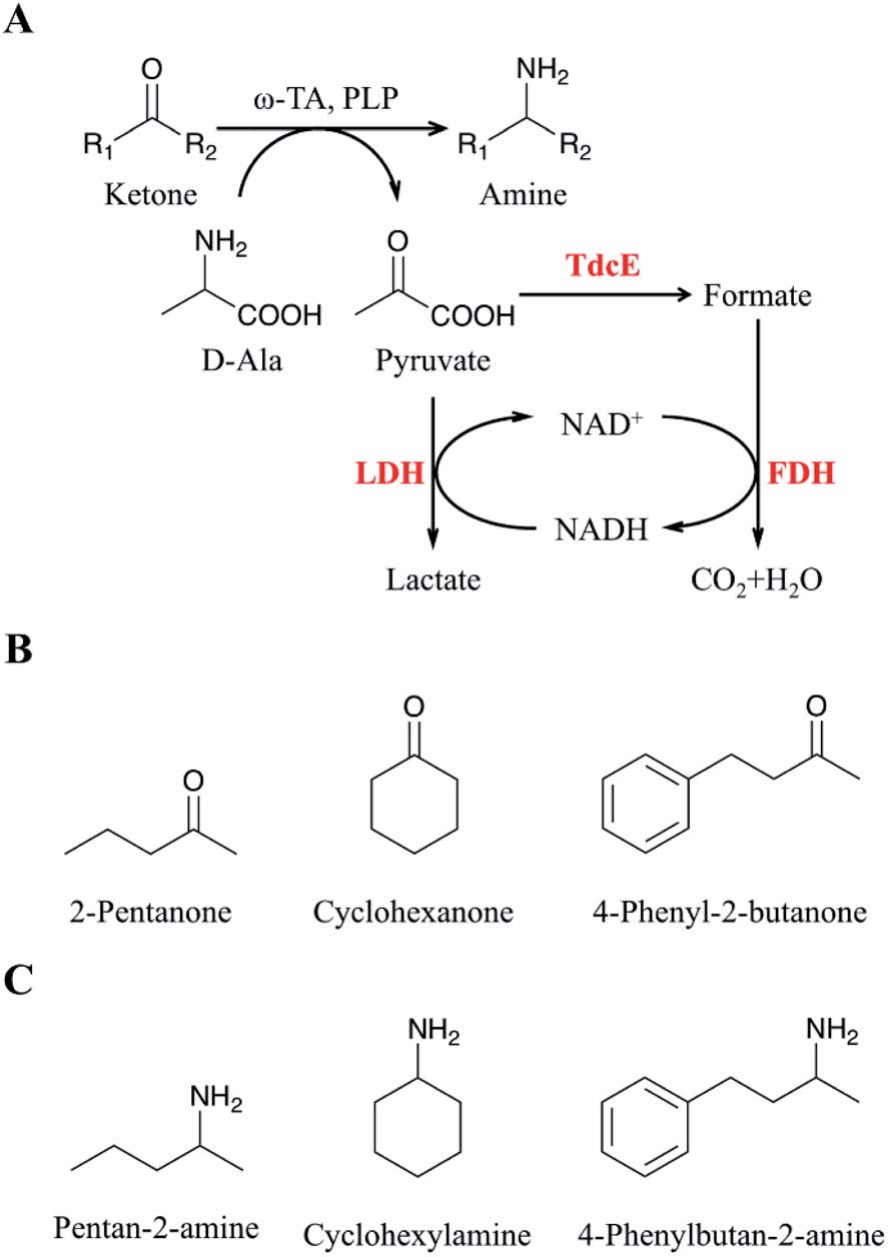
Asymmetric amination of ketones using ω-transaminase. (A) Pyruvate is removed by the TdcE/FDH/LDH system. Ketones: substrates for transamination reaction; amine: products for transamination reaction; R_1_ and R_2_: methyl, propyl, cyclohexyl, ethylphenyl; ω-TA: ω-transaminase; PLP: pyridoxal phosphate; LDH: lactate dehydrogenase; TdcE: formate acetyltransferase; FDH: formate dehydrogenase. (B) Substrates for transamination reaction in this study. (C) Products for transamination reaction in this study.

Here, we developed a novel system to enhance the conversion of ketones to amines. Firstly, we overexpressed four formate acetyltransferases (*i.e.*, Ybiw, PflB, PflD and TdcE) derived from *Escherichia coli* MG1655 to convert pyruvate to formate, and we found TdcE had the highest specific activity. Formate dehydrogenase (FDH) was introduced to decompose formate and produce NADH. LDH was introduced to recycle NADH and remove pyruvate. Thus, the ArR-ωTA/TdcE/FDH/LDH system was constructed for transamination reaction (Fig. 1A). The substrates and products for the transamination reaction in this study were shown in Fig. 1B and C, respectively. Secondly, we optimized the reaction conditions, including d-alanine, DMSO, and PLP with different concentration of 2-pentanone (as a model substrate) to increase the conversion of ketones to amines. Thirdly, the conversions of 2-pentanone, 4-phenyl-2-butanone and cyclohexanone by using the ArR-ωTA/TdcE/FDH/LDH system were 84.5%, 98.2% and 79.3%, respectively. Besides, the pyruvate to formate pathway (*i.e.* TdcE/FDH) dominates the by-product pyruvate removal in the transamination reaction. This work demonstrated that the ArR-ωTA/TdcE/FDH/LDH system could effectively convert ketones to amines.

## 2. Materials and methods

### 2.1 Gene synthesis and plasmid construction

FDH is from *Candida boidinii* (Sigma). (*R*)-Selective ω-transaminase derived from *Arthrobacter* sp. (NCBI GenBank: BAK39753.1) was identified from NCBI. Its amino acid sequences were optimized in the *E. coli* strain BL21 (DE3) in JCAT, and the restriction sites of NdeI, SpeI, HindIII and XbaI were avoided in the optimal sequence. The *gdh* gene (NCBI Gene ID: 938377) originated from *Bacillus subtilis* was obtained in the same way as the *arR-ωTA* gene. The *arR-ωTA* gene and *gdh* gene were *in vitro* synthesized. The *ybiw* (NCBI Gene ID: 945444), *pflB* (NCBI Gene ID: 945514), *pflD* (NCBI Gene ID: 948454), *tdcE* (NCBI Gene ID: 947623) and *ldh* (NCBI GenBank: APQ21713.1) were amplified from the genomic DNA of the *E. coli* MG1655 through PCR with primers. The gene of green fluorescent protein (GFP) was from our research lab. Tables S1 and S2† list all primers utilized and synthesized gene sequences in this research, respectively.

**Fig. 2:**
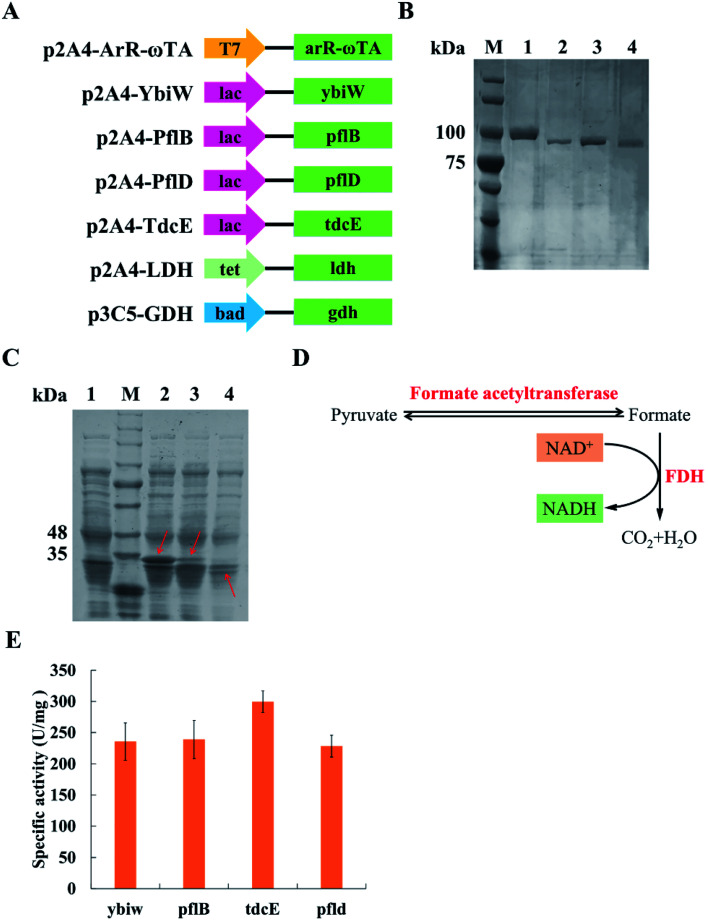
SDS analysis and the analysis of four formate acetyltransferases. (A) Schematic diagram of plasmids used in this study. Four promoters: T7, lac, tet and bad; the gene of (*R*)-selective ω-transaminase from *Arthrobacter* sp.: *arR-ωTA*; four formate acetyltransferase genes: *ybiW*, *pflB*, *pflD* and *tdcE*; the gene of lactate dehydrogenase (LDH): *ldh*; the gene of glucose dehydrogenase (GDH): *gdh*. (B) SDS-PAGE analysis of purified formate acetyltransferases. M, protein marker; lane 1, YbiW; lane 2, PflB; lane 3, TdcE; lane 4, PflD. (C) SDS-PAGE analysis. M, protein marker; lane 1, the blank control; lane 2, ArR-ωTA; lane 3, LDH; lane 4, GDH. (D) Mechanism of measurement of formate acetyltransferases activity. FDH, formate dehydrogenase. (E) Specific activity of the four formate acetyltransferases. IPTG was added to a final concentration of 1 mM. Results are represented as mean ± SD of three replicates.

ArR-ωTA, Ybiw, PflB, PflD, TdcE, LDH and GFP were constructed in the p2A4 vector containing pBR322 origin. GDH and GFP were constructed in the p3C5 vector containing p15A origin. All genes were inserted into vectors by biobrick. All the plasmids utilized in this research are shown in Table 1. *E. coli* BL21 (DE3) was utilized for gene expression. Fig. 2A shows schematic of plasmids used in this research.

**Table 1:** Plasmids used in this study

Plasmids	Characteristics	Source
p2A4	Amp^R^	Our lab
p2A4T7	p2A4 derivate, P_T7_	This work
p2A4T7-ArR-ωTA	ArR-ωTA gene in p2A4T7	This work
p2A4tet	p2A4 derivate, P_tet_	This work
p2A4tet-LDH	LDH gene in p2A4tet-LDH	This work
p2A4Lac	p2A4 derivate, P_Lac_	Our lab
p2A4Lac-Ybiw	Ybiw gene in p2A4Lac	This work
p2A4Lac-pflB	pflB gene in p2A4Lac	This work
p2A4Lac-pflD	pflD gene in p2A4Lac	This work
p2A4Lac-tdcE	tdcE gene p2A4Lac	This work
p3C5	Cm^R^	Our lab
p3C5BAD	p3C5 derivate, P_BAD_	This work
p3C5BAD-GDH	GDH gene in p3C5BAD	This work

### 2.2 Gene expression in *E. coli*

The transformed strains were cultured in LB liquid medium containing appropriate antibiotics (100 mg ampicillin per L or 34 mg chloramphenicol per L), and cultivation for overnight at 37 °C and 220 rpm. The seed cultures were diluted 1 : 100 in fresh Terrific Broth medium containing 1% (w/v) glucose and appropriate antibiotics, and they were incubated at 37 °C. When the optical density at 600 nm (OD_600_) reached approximately 0.6, the temperature was decreased to 25 °C with shaking at 200 rpm. Then, a final concentration of 1 mM isopropyl-β-d-thiogalactopyranoside (IPTG), 10 mM aTc or 1 mM l-arabinose was added, and cells were cultured at 25 °C for 16 h.

### 2.3 Representative example for amination

Cells containing ArR-ωTA, TdcE, LDH or GDH were collected by centrifugation at 6000 rpm for 10 min at 4 °C, respectively. Then, cells were suspended in PBS buffer (100 mM, pH 7.5) for the standardization of samples (OD_600_ = 120) and lysed through sonication over ice for 30 min with 5 s pulses at 10 s intervals. Cells lysates were regarded as crude products. All reactions were carried out at 30 °C and 180 rpm for 24 h in PBS buffer (1 mL, 100 mM, pH 7.5, 1.5 mM PLP, 1 mM NAD^+^). The ArR-ωTA/LDH/GDH system: 500 μL crude product of ArR-ωTA, 120 μL crude product of LDH, 120 μL crude product of GDH, d-alanine (250 mM, 22.3 mg), ketone (25 mM), glucose (150 mM) and 30% (v/v) DMSO were added. The ArR-ωTA/TdcE/FDH/LDH system: 500 μL crude product of ArR-ωTA, 120 μL crude product of LDH, 120 μL crude product of TdcE, FDH (10 U), d-alanine (250 mM, 22.3 mg), ketone (25 mM), CoA (0.1 mM) and 30% (v/v) DMSO were added. Aqueous NaOH (200 μL, 10 N) was added to stop the reaction. Ethyl acetate (500 μL, twice) was utilized to extract the mixture, and Na_2_SO_4_ was used to dry the organic phases. The conversion of ketones to amines was measured by gas chromatograph (GC).

### 2.4 GC method for conversion and optical purity determination

The conversions were analysed by GC-MS on TG-5MS column (30 m × 0.32 mm × 0.25 μm; Shimadzu). The GC program parameters included the following: injector 250 °C; constant flow 1.8 mL min^−1^; temperature program 40 °C/hold 2 min; 80 °C/rate 5 °C per min/hold 2 min; 250 °C/rate 20 °C per min/hold 10 min. GC analyses and mass spectrometry analyses in this study are shown in Table S3 and Fig. S1,† respectively. The external standard method was utilized in gas chromatography experiments and the standard curves of three substrates are shown in Fig. S2.†

The optical purity of reaction products were determined by GC on CP-Chirasil-Dex CB column (25 m × 0.32 mm × 0.25 μm; Agilent) after derivatization. Derivative method and analytical method are from Mutti *et al.*^33^ The chiral analyses of products are shown in Table S4.†

### 2.5 SDS-PAGE

Collecting of cells was performed *via* centrifugation at 6000 rpm and lysed through sonication on ice. The soluble fractions were extracted from supernatant at 10 000 rpm for 10 min at 4 °C. Protein samples were analysed on 10% acrylamide gels.

### 2.6 Purification of formate acetyltransferase

Collecting of cells was performed *via* centrifugation at 6000 rpm for 10 min at 4 °C and suspended in phosphate buffer (100 mM, pH 7.5). Cell lysed through sonication over ice for 30 min with 5 s pulses at 10 s intervals. The soluble fraction of cell lysates was separated by centrifugation (10 000 rpm for 20 min at 4 °C). The formate acetyltransferase were carried out by Ni-Sepharose purification (Thermo Fisher Scientific). The purified proteins were utilized for SDS-PAGE analysis and measurement of enzyme activity.

### 2.7 Measurement of formate acetyltransferases activity

The enzyme activities of the four formate acetyltransferases were determined by the double enzymatic method,^34^ based on the following reactions:
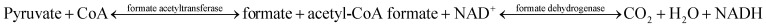


The reactions were carried out in total volume of 1 mL, with the compositions of sodium pyruvate (20 mM), CoA (0.1 mM), NAD^+^ (1 mM), FDH (1 U), PBS buffer (100 mM, pH 7.5) and purified formate acetyltransferase (0.69 mg mL^−1^). The mixture was shaken at 30 °C for 10 s, and the concentration of NADH was measured as OD_340_. The curves of OD_340_ with time are shown in Fig. S3.†

## 3. Results and discussion

### 3.1 Overexpression and analysis of formate acetyltransferases

Pyruvate can be catalysed to produce lactate and formate in *E. coli*. Formate acetyltransferase, also known as pyruvate formate lyase, is an enzyme to transform pyruvate into formate. There are four formate acetyltransferases derived from *E. coli* MG1655 (*i.e.*, Ybiw, PflB, PflD and TdcE) catalysing the reaction from pyruvate to formate. In this study, we constructed plasmids for these four genes (Fig. 2A) and confirmed their soluble expression (Fig. 2B). The catalytic activity of four formate acetyltransferases were compared through the formation of formate. Considering the reversible reaction between pyruvate and formate, FDH was introduced to decompose formate and produce NADH (Fig. 2D). Thus, we tested these four enzymes activities by determining the concentration of NADH at OD_340_. We demonstrated that the overexpression of TdcE enabled the highest specific activity (299.3 U mg^−1^ purified protein) in comparison to the other three formate acetyltransferases (Fig. 2E). Therefore, TdcE was selected as the enzyme to catalyse the reaction from pyruvate to formate for subsequent experiments. Furthermore, TdcE/FDH was regarded as the formate pathway to remove pyruvate. Considering the recycle of co-factor NADH, LDH was introduced to recover NAD^+^. In addition, LDH was regarded as the lactate pathway to remove pyruvate. Thus, a novel multi-enzyme system, *i.e.*, the ω-TA/TdcE/FDH/LDH system, was established for transamination reaction (Fig. 1A).

In order to determine the expression level of promoters, we constructed four inducible promoters (*i.e.*, P_T7_, P_lac_, P_tet_, P_bad_) to drive the expression of *gfp* gene, respectively (Fig. S4†). The expression levels of TdcE, LDH and GDH should be similar, thus avoiding the effect of enzyme dosages on the experimental results. The results showed that the GFP/OD_600_ values of P_lac_, P_tet_ and P_bad_ were all about 20 000, when the inducer concentrations were 1 mM IPTG, 10 mM aTc and 1 mM l-arabinose, respectively. Therefore, 1 mM IPTG, 10 mM aTc and 1 mM l-arabinose were selected for further studies. The soluble fractions of cells containing ArR-ωTA, LDH or GDH were analysed by SDS-PAGE (Fig. 2C).

### 3.2 Optimization of the condition of transamination reaction

Reaction condition is a key factor in the transamination reaction. We investigated the effects of d-alanine, DMSO, substrate concentration and PLP on the conversion of ketones to amines. 2-Pentanone was used as a model substrate in the optimization of reaction conditions.

**Fig. 3:**
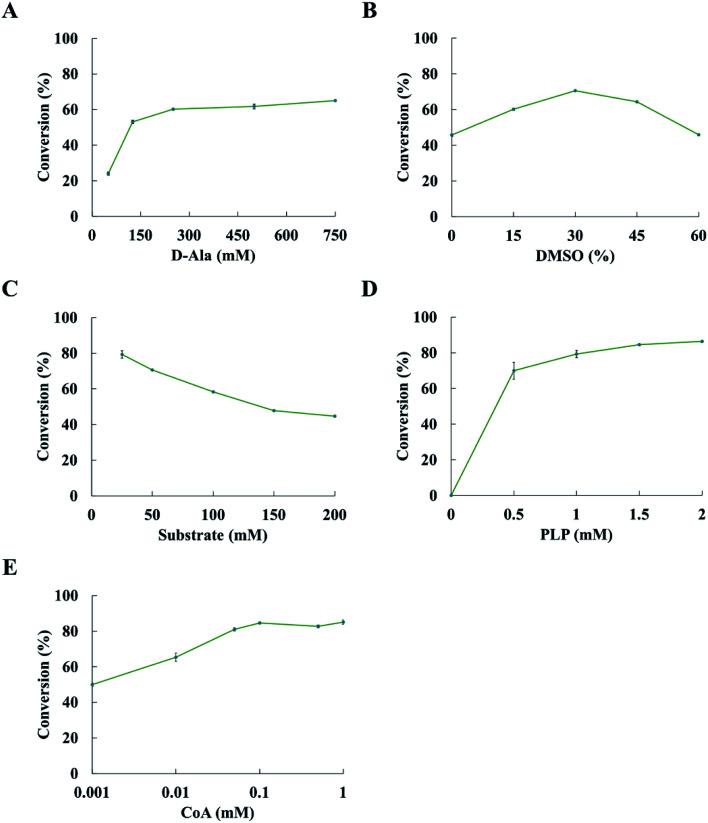
Optimization of the condition of transamination reaction. (A–D) Effect of varied d-alanine, DMSO, substrate and PLP concentrations on the asymmetric amination of 2-pentanone catalyzed by the ArR-ωTA/TdcE/FDH/LDH system, respectively. (E) Effect of different CoA concentrations on the asymmetric amination of 2-pentanone catalysed by the ArR-ωTA/TdcE/FDH/LDH system. Conditions: 2-pentanone (25 mM), 30% (v/v) DMSO, d-alanine (250 mM), PBS buffer (pH 7.5, 100 mM), PLP (1.5 mM), NAD^+^ (1 mM), FDH (10 U), ArR-ωTA (500 μL), TdcE (120 μL), LDH (120 μL), shaking at 30 °C and 180 rpm for 24 h. Results are represented as mean ± SD of three replicates.

The conversion was improved with the concentration of d-alanine increasing from 50 mM to 250 mM; however, there was a slight increase in conversion when the concentration of d-alanine was more than 250 mM (Fig. 3A). The conversion could increase up to 60.2% at 250 mM d-alanine. Thus, 250 mM d-alanine was selected for further experiments. When the DMSO concentration increased from 0 to 30% (v/v), the conversion was also enhanced. While the conversion decreases when the DMSO concentration was over 30% (v/v). Optimization of the DMSO concentration led to 70.6% conversion at 30% (v/v) DMSO (Fig. 3B). It is noted that the conversion was decreased with the concentration of substrate increases at 30% (v/v) DMSO and 250 mM d-alanine concentration (Fig. 3C). The conversion reached 79.3% at 25 mM substrate concentration. The conversion of ketone to amine was increased when more PLP was added, while the concentration of PLP has minor positive impact on the conversion at the PLP concentration above 1.5 mM (Fig. 3D). Upon optimization of reaction conditions, the conversion of 2-pentanone to pentan-2-amine reached 84.5%. Thus, subsequent experiments were performed at 250 mM d-alanine, 30% (v/v) DMSO, 25 mM substrate and 1.5 mM PLP.

### 3.3 Analysis the asymmetric amination of the ArR-ωTA/TdcE/FDH/LDH system

**Fig. 4:**
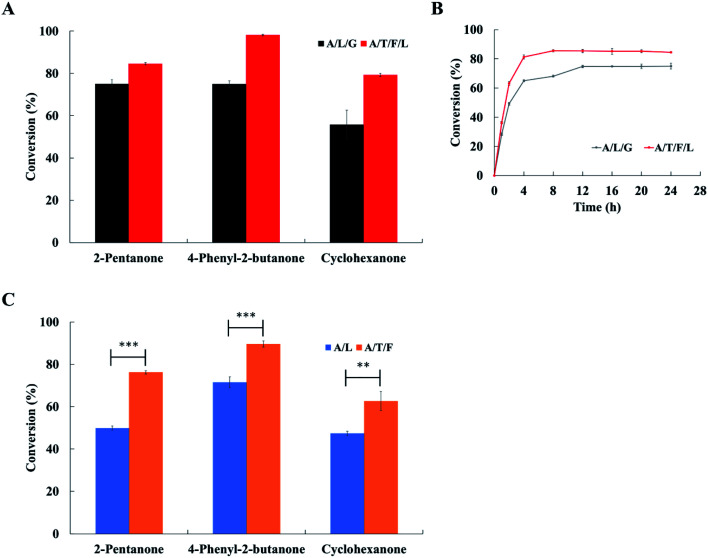
Asymmetric amination of ketones using ArR-ωTA. (A) Effect of different systems (the ArR-ωTA/LDH/GDH system, and the ArR-ωTA/TdcE/FDH/LDH system) on the asymmetric amination of ketones. A/L/G, the ArR-ωTA/LDH/GDH system; A/T/F/L, the ArR-ωTA/TdcE/FDH/LDH system. ArR-ωTA/LDH/GDH system, conditions: substrate (25 mM), 30% (v/v) DMSO, d-alanine (250 mM), glucose (150 mM), PBS buffer (pH 7.5, 100 mM), PLP (1.5 mM), NAD^+^ (1 mM), ArR-ωTA (500 μL), LDH (120 μL), GDH (120 μL), shaking at 30 °C and 180 rpm for 24 h; ArR-ωTA/TdcE/FDH/LDH system, conditions: substrate (25 mM), 30% (v/v) DMSO, d-alanine (250 mM), PBS buffer (pH 7.5, 100 mM), PLP (1.5 mM), NAD^+^ (1 mM), CoA (0.1 mM), FDH (10 U), ArR-ωTA (500 μL), TdcE (120 μL), LDH (120 μL), shaking at 30 °C and 180 rpm for 24 h. (B) The conversion of 2-pentanone to pentan-2-amine at different reaction times. A/L/G, the ArR-ωTA/LDH/GDH system; A/T/F/L, the ArR-ωTA/TdcE/FDH/LDH system. ArR-ωTA/LDH/GDH system, conditions: substrate (25 mM), 30% (v/v) DMSO, d-alanine (250 mM), glucose (150 mM), PBS buffer (pH 7.5, 100 mM), PLP (1.5 mM), NAD^+^ (1 mM), ArR-ωTA (500 μL), GDH (120 μL), LDH (120 μL); ArR-ωTA/TdcE/FDH/LDH system, conditions: substrate (25 mM), 30% (v/v) DMSO, d-alanine (250 mM), PBS buffer (pH 7.5, 100 mM), PLP (1.5 mM), NAD^+^ (1 mM), CoA (0.1 mM), FDH (10 U), ArR-ωTA (500 μL), TdcE (120 μL), LDH (120 μL). (C) Effect of two pyruvate removal pathways (the LDH pathway, pyruvate to lactate; the TdcE/FDH pathway, pyruvate to formate) on the asymmetric amination of ketones. A/L, the ArR-ωTA/LDH system; A/T/F, the ArR-ωTA/TdcE/FDH system. ArR-ωTA/LDH system, conditions: substrate (25 mM), 30% (v/v) DMSO, d-alanine (250 mM), PBS buffer (pH 7.5, 100 mM), PLP (1.5 mM), NADH (1 mM), CoA (0.1 mM), ArR-ωTA (500 μL), LDH (120 μL), shaking at 30 °C and 180 rpm for 24 h; ArR-ωTA/TdcE/FDH system, conditions: substrate (25 mM), 30% (v/v) DMSO, d-alanine (250 mM), PBS buffer (pH 7.5, 100 mM), PLP (1.5 mM), NAD^+^ (1 mM), CoA (0.1 mM), FDH (10 U), ArR-ωTA (500 μL), TdcE (120 μL), shaking at 30 °C and 180 rpm for 24 h. Results are represented as mean ± SD of three replicates. **p < 0.01; ***p < 0.001.

Three substrates (*i.e.*, 2-pentanone, 4-phenyl-2-butanone and cyclohexanone) were examined to compare the conversion of ketones to amines obtained with both the ArR-ωTA/TdcE/FDH/LDH system and the ArR-ωTA/LDH/GDH system. By using the ArR-ωTA/TdcE/FDH/LDH system, the conversion of 2-pentanone to pentan-2-amine was increased from 75.1% (by the ArR-ωTA/LDH/GDH system) to 84.5%, the conversion of 4-phenyl-2-butanone to 4-phenylbutan-2-amine was increased from 74.9% to 98.2%, and the conversion of cyclohexanone to cyclohexylamine was increased from 55.8% to 79.3%, respectively (Fig. 4A). Besides, the conversion of 2-pentanone to pentan-2-amine and the conversion of 4-phenyl-2-butanone to 4-phenylbutan-2-amine with the ArR-ωTA/LDH/GDH system reported in the literature were only 67% and 82%, respectively.^33^ The enantiomeric excess of reaction products was shown in Table 2. It is remarkable that the ArR-ωTA/TdcE/FDH/LDH system could efficiently synthesize chiral amines. Moreover, the ArR-ωTA/TdcE/FDH/LDH system does not need to be provided with co-substrate (*i.e.*, glucose).

**Table 2:** Effect the ArR-ωTA/LDH/GDH system and the ArR-ωTA/TdcE/FDH/LDH system on the asymmetric amination of ketones

Entry	Substrate	A/L/G system^a^		A/T/F/L system^b^	
		Conversion [%]	ee_amine_^c^ [%]	Conversion [%]	ee_amine_^c^ [%]
1	2-Pentanone	75.1	>99(*R*)	84.5	>99(*R*)
2	4-Phenyl-2-butanone	74.9	>99(*R*)	98.2	>99(*R*)
3	Cyclohexanone	55.8	n.a.^d^	79.3	n.a.^d^

a A/L/G, the ArR-ωTA/LDH/GDH system. Conditions: substrate (25 mM), 30% (v/v) DMSO, d-alanine (250 mM), glucose (150 mM), PBS buffer (pH 7.5, 100 mM), PLP (1.5 mM), NAD^+^ (1 mM), ArR-ωTA (500 μL), LDH (120 μL), GDH (120 μL), shaking at 30 °C and 180 rpm for 24 h.

b A/T/F/L, the ArR-ωTA/TdcE/FDH/LDH system. Conditions: substrate (25 mM), 30% (v/v) DMSO, d-alanine (250 mM), PBS buffer (pH 7.5, 100 mM), PLP (1.5 mM), NAD^+^ (1 mM), CoA (0.1 mM), FDH (10 U), ArR-ωTA (500 μL), TdcE (120 μL), LDH (120 μL), shaking at 30 °C and 180 rpm for 24 h.

c Optical purity of reaction products was determined by GC.

d n.a.: not applicable.

2-Pentanone was used as a model substrate to compare the conversion of these two systems at different reaction times. The conversion with the ArR-ωTA/TdcE/FDH/LDH system reached the maximum value within 4 to 8 h, while the conversion with the ArR-ωTA/LDH/GDH system reached the maximum value within 8 to 12 h (Fig. 4B). Compared with the ArR-ωTA/LDH/GDH system, the ArR-ωTA/TdcE/FDH/LDH system led to a shorter reaction time and a higher conversion. The ArR-ωTA/TdcE/FDH/LDH system has two enzymes, TdcE and LDH, to remove the by-product pyruvate, thereby shifting the equilibrium toward amine synthesis. Furthermore, FDH and LDH in the ArR-ωTA/TdcE/FDH/LDH system could not only recycle the redox cofactor (*i.e.*, NAD^+^ and NADH), but also promote the removal of pyruvate by decomposing formate.

De-acetylation of pyruvate to formate catalysed by TdcE was accompanied by the conversion of CoA to acetyl CoA. Thus, regeneration of CoA from acetyl CoA would be a crucial step for the feasibility of ArR-ωTA/TdcE/FDH/LDH cascade. Acetyl-CoA reacted with oxaloacetate to give CoA and citrate in the tricarboxylic acid cycle. The crude enzyme preparations contained GltA (citrate synthase) that catalysed this reaction, thereby regenerating CoA from acetyl-CoA. We also performed experiments on varied concentrations of CoA in the ArR-ωTA/TdcE/FDH/LDH system, in which 2-pentanone was used as a model substrate, as shown in Fig. 3E. The results showed that the conversion of 2-pentanone to pentan-2-amine barely increased when the concentration of CoA was more than 0.1 mM. It proved that 0.1 mM CoA was sufficient, and the regeneration of CoA from acetyl CoA was feasible in the ArR-ωTA/TdcE/FDH/LDH system.

In order to investigate which of the two pyruvate removal pathways dominates, three substrates were tested to compare the conversion of ketones to amines obtained with the ArR-ωTA/LDH system and the ArR-ωTA/TdcE/FDH system. 1 mM NADH was added to the ArR-ωTA/LDH system, and 1 mM NAD^+^ was added to the ArR-ωTA/TdcE/FDH system. Under the ArR-ωTA/TdcE/FDH system, the conversions of 2-pentanone, 4-phenyl-2-butanone and cyclohexanone were 76.3%, 89.6% and 62.7%, respectively. However, the conversions of 2-pentanone, 4-phenyl-2-butanone and cyclohexanone *via* using the ArR-ωTA/LDH system were 49.9%, 71.5% and 47.4%, respectively (Fig. 4C). The experimental results showed that the ArR-ωTA/TdcE/FDH system led to a higher conversion of ketones to amines than that of the ArR-ωTA/LDH system. Furthermore, it illustrated that the formate pathway (*i.e.* TdcE/FDH) dominates the by-product pyruvate removal in the transamination reaction.

## 4. Conclusions

We compared specific activities of four formate acetyltransferases and constructed a novel system by combination of ArR-ωTA, TdcE, FDH and LDH. Optimization of reaction conditions of d-alanine, DMSO, substrate concentration and PLP further increased the conversion of ketones to amines. By using the ArR-ωTA/TdcE/FDH/LDH system, the conversions of 2-pentanone, 4-phenyl-2-butanone and cyclohexanone were 84.5%, 98.2% and 79.3%, respectively. Moreover, we found that the by-product pyruvate removal in the transamination reaction was dominated by the pyruvate to formate pathway (*i.e.* TdcE/FDH). The ArR-ωTA/TdcE/FDH/LDH system was suitable for multiple types of substrates, such as aromatic substrates (*e.g.*, 4-phenyl-2-butanone), aliphatic substrates (*e.g.*, 2-pentanone) and alicyclic substrates (*e.g.*, cyclohexanone). In conclusion, the ArR-ωTA/TdcE/FDH/LDH system was confirmed to be an efficient system for the removal of pyruvate, thus facilitating the conversion of transamination reaction.

There is too an increase in the complexity so there are more enzymes involved than that for reported system (*i.e.*, ArR-ωTA/LDH/GDH three enzymes' system). However, the four enzymes in our system (*i.e.*, ArR-ωTA/TdcE/FDH/LDH four enzymes' system) could be co-expressed in one engineered strain of *E. coli*, which makes the enzymes synthesis and separation relatively cheap. Therefore, there is a negligible increase in the enzymes production cost in our system compared with the reported system. In addition, the conversion with our system reached maximum within 4 to 8 h, while the conversion with the reported system reached maximum within 8 to 12 h. Compared with the reported system, our system led to a shorter reaction time and ∼20% increase in the conversion. Thus, our system has advantages in the production cost.

## Conflicts of interest

There are no conflicts of interest to declare.

## Supplementary Material

RA-010-D0RA06140A-s001
