# Supplementary material for: Multi-enzyme pyruvate removal system to enhance (R)-selective reductive amination of ketones
Source: RSC Adv. 2020 Aug 5;10(48):28984–91. doi: 10.1039/d0ra06140a (PMC9055928; doi:10.1039/d0ra06140a)
Supplement: RA-010-D0RA06140A-s001 [file RA-010-D0RA06140A-s001.pdf]

Electronic Supplementary Material (ESI) for RSC Advances.
This journal is © The Royal Society of Chemistry 2020

Supporting Information (SI)

Multi-enzyme pyruvate removal system to enhance (*R*)-selective reductive amination of ketone

Jinhua Zhang, Yanshu Zhao, Chao Li and Hao Song*

Frontier Science Center for Synthetic Biology and Key Laboratory of Systems Bioengineering (MOE), School of Chemical Engineering and Technology, Tianjin University, Tianjin, 300350, P. R. China

* Corresponding author: H. Song. E-mail: hsong@tju.edu.cn. Tel: 86-18722024233.

Table S1. Sequences of the primers used in this study

Name	Sequence (5’-3’)
ArR-ωTA-F	CATATGGCTTTCTCTGCTGACACCTCT
ArR-ωTA-R	AATACTAGTTAGTACTGAACCGGGGTC
LDH-F	GGAATTCCATATGATGAAACTCGC
LDH-R	ACTAGTTTAAACCAGTTCGTTCGGG
GDH-F	GGAATTCCATATGCATCACCATCATCACCACTACCCGGAC
GDH-R	GGACTAGTTTACCACGACCAGCCT
Ybiw-F	GGAATTCCATATGCATCACCATCATCACCACACCACACTGAAACTG
Ybiw-R	GGACTAGTTTACAGCATATGTTCAGTACGG
PflB-F	GGAATTCCATATGCATCACCATCATCACCACATCCGAGCTTA
PflB-R	GGACTAGTTACATAGATTGAGTGAAGG
PflD-F	GGAATTCCATATGCATCACCATCATCACCACACGAATCGTATCTC
PflD-R	GGACTAGTTACAGCTGATGCGCT
TdcE-F	GGAATTCCATATGCATCACCATCATCACCACAAGGTAGATATTGA
TdcE-R	GGACTAGTTCAGAGCGCCTGGGTAA

Table S2. Synthesized gene sequences in this study

Genes	Sequences
ArR-ωTA	CATATGGCTTTCTCTGCTGACACCTCTGAAATCGTTTACACCCACGACACCGGTCTGGACTACATCACC<br>TACTCTGACTACGAACTGGACCCGGCTAACCCGCTGGCTGGTGGTGCTGCTTGGATCGAAGGTGCTTT<br>CGTTCCGCCGTCTGAAGCTCGTATCTCTATCTTCGACCAGGGTTACCTGCACTCTGACGTTACCTACAC<br>CGTTTTCCACGTTTGGAACGGTAACGCTTTCCGTCTGGACGACCACATCGAACGTCTGTTCTCTAACGC<br>TGAATCTATGCGTATCATCCCGCCGCTGACCCAGGACGAAGTTAAAGAAATCGCTCTGGAACTGGTTG<br>CTAAAACCGAACTGCGTGAAGCGTTTGTATCTGTAAGCATCACCCGTGGTTACTCTTCTACCCCGGGTG<br>AACGTGACATCACCAAACACCGTCCGCAGGTTTACATGTACGCTGTTCCGTACCAGTGGATCGTTCCG<br>TTCGACCGTATCCGTGACGGTGTTCACGCTATGGTTGCTCAGTCTGTTCGTCGTACCCCGCGTTCTTCTA<br>TCGACCCGCAGGTTAAAAACTTCCAGTGGGGTGACCTGATCCGTGCTGTTCAGGAAACCCACGACAGG<br>GGCTTCGAAGCTCCGCTCCTGCTGGACGGTGACGGTCTGCTGGCTGAAGGTTCTGGTTTCAACGTTGTT<br>GTTATCAAAGACGGTGTTGTTCGTTCTCCGGGTCGTGCTGCTCTGCCGGGTATCACCCGTAAAACCGTT<br>CTGGAAATCGCTGAATCTCTGGGTCACGAAGCTATCCTGGCTGACATCACCCTGGCTGAACTGCTGGA<br>CGCTGACGAAGTTCTGGGTTGCACCACAGCTGGCGGTGTCTGGCCGTTCGTCTCTGTTGACGGTAACCC<br>GATCTCTGACGGTGTTCCGGGTCCGGTTACCCAGTCTATCATCCGTCGTTACTGGGAACTGAACGTTGA<br>ATCTTCTTCTCTGCTGACCCCGGTTCAGTACTAACTAGTATTCAAGCTT
gfp	ATGCGTAAAGGAGAAGAACTTTTCACTGGAGTTGTCCCAATTCTTGTTGAATTAGATGGTGATGTTAAT<br>GGGCACAAATTTTCTGTCAGTGGAGAGGGTGAAGGTGATGCAACATACGGAAAACTTACCCTTAAATT<br>TATTTGCACTACTGGAAAACTACCTGTTCCGTGGCCAACACTTGTCACTACTTTCGGTTATGGTGTTCA<br>ATGCTTTGCGAGATACCCAGATCACATGAAACAGCATGACTTTTTCAAGAGTGCCATGCCCGAAGGTT<br>ATGTACAGGAAAGAACTATATTTTTCAAAGATGACGGGAACTACAAGACACGTGCTGAAGTCAAGTTT<br>GAAGGTGATACCCTTGTTAATAGAATCGAGTTAAAAGGTATTGATTTTAAAGAAGATGGAAACATTCT<br>TGGACACAAATTGGAATACAACTATAACTCACACAATGTATACATCATGGCAGACAAACAAAAGAAT<br>GGAATCAAAGTTAACTTCAAAATTAGACACAACATTGAAGATGGAAGCGTTCAACTAGCAGACCATT<br>ATCAACAAAATACTCCAATTGGCGATGGCCCTGTCCTTTTACCAGACAACCATTACCTGTCCACACAAT<br>CTGCCCTTTCGAAAGATCCCAACGAAAAGAGAGACCACATGGTCCTTCTTGAGTTTGTAACAGCTGCT<br>GGGATTACACATGGCATGGATGAACTATACAAATAATAA

ldh	GGAATTCCATATGATGAAACTCGCCGTTTATAGCACAAAACAGTACGACAAGAAGTACCTGCAACAG<br>GTGAACGAGTCCTTTGGCTTTGAGCTGGAATTTTTTGACTTTCTGCTGACGGAAAAAACCGCTAAAACT<br>GCCAATGGCTGCGAAGCGGTATGTATTTTCGTAAACGATGACGGCAGCCGCCCGGTGCTGGAAGAGCT<br>GAAAAAGCACGGCGTTAAATATATCGCCCTGCGCTGTGCCGGTTTCAATAACGTCGACCTTGACGCGG<br>CAAAAGAACTGGGGCTGAAAGTAGTCCGTGTTCCAGCCTATGATCCAGAGGCCGTTGCTGAACACGCC<br>ATCGGTATGATGATGACGCTGAACCGCCGTATTCACCGCGCGTATCAGCGTACCCGTGATGCTAACTT<br>CTCTCTGGAAGGTCTGACCGGCTTTACTATGTATGGCAAAACGGCAGGCGTTATCGGTACCGGTAAAA<br>TCGGTGTGGCGATGCTGCGCATTCTGAAAGGTTTTGGTATGCGTCTGCTGGCGTTCGATCCGTATCCAA<br>GTGCAGCGGCGCTGGAACTCGGTGTGGAGTATGTCGATCTGCCAACCCTGTTCTCTGAATCAGACGTT<br>ATCTCTCTGCACTGCCCGCTGACACCGGAAAACTATCATCTGTTGAACGAACCGCCTTCGAACAGATG<br>AAAAATGGCGTGATGATCGTCAATACCAGTCGCGGTGCATTGATTGATTCTCAGGCAGCAATTGAAGC<br>GCTGAAAAATCAGAAAATTGGTTCGTTGGGTATGGACGTGTATGAGAACGAACGCGATCTATTCTTTG<br>AAGATAAATCCAACGACGTGATCCAGGATGACGTATTCCGTCGCCTGTCTGCCTGCCACAACGTGCTG<br>TTTACCGGGCACCAGGCATTCCTGACAGCAGAAGCTCTGACCAGTATTTCTCAGACTACGCTGCAAAA<br>CTTAAGCAATCTGGAAAAAGGCGAAACCTGCCCGAACGAACTGGTTTAAACTAGTCC
gdh	GGAATTCCATATGCATCACCATCATCACCACTACCCGGACCTGAAAGGTAAAGTTGTTGCTATCACCG<br>GTGCTGCTTCTGGTCTGGGTAAAGCTATGGCTATCCGTTTCGGTAAAGAACAGGCTAAAGTTGTTATCA<br>ACTACTACTCTAACAAACAGGACCCGAACGAAGTTAAAGAAGAAGTTATCAAAGCTGGTGGTGAAGC<br>TGTTGTTGTTCAGGGTGACGTTACCAAAGAAGAAGACGTTAAAAACATCGTTCAGACCGCTATCAAAG<br>AATTTGGTACCCTGGACATCATGATCAACAACGCTGGTCTGGAAAACCCGGTTCCGTCTCACGAAATG<br>CCGCTGAAAGACTGGGACAAAGTTATCGGTACCAACCTGACCGGTGCTTTCCTGGGTTCTCGTGAAGC<br>TATCAAATACTTCGTTGAAAACGACATCAAAGGTAACGTTATCAACATGTCTTCTGTTCACGAAGTTAT<br>CCCGTGGCCGCTGTTCGTTCACTACGCTGCTTCTAAAGGTGGTATCAAACTGATGACCGAAACCCTGG<br>CTCTGGAATACGCTCCGAAAGGTATCCGTGTTAACAACATCGGTCCGGGTGCTATCAACACCCCGATC<br>AACGCTGAAAAATTCGCTGACCCGAAACAGAAAGCTGACGTTGAATCTATGATCCCGATGGGTTACAT<br>CGGTGAACCGGAAGAAATCGCTGCTGTTGCTGCTTGGCTGGCTTCTAAAGAAGCTAGTTACGTTACCG<br>GTATCACCCTGTTCGCTGACGGTGGTATGACCCAGTACCCGTCTTTCCAGGCTGGTCGTGGTAAACTAG<br>TCC

ybiw	GAATTCCATATGCATCACCATCATCACCACACCACACTGAAACTGGACACGCTCAGCGACCGCATTAA<br>AGCGCACAAAAATGCGCTGGTGCATATTGTGAAACCGCCAGTCTGTACCGAGCGCGCGCAGCACTATA<br>CCGAGATGTATCAACAACATCTCGATAAGCCGATCCCGGTACGTCGCGCGCTGGCACTGGCGCATCAC<br>CTGGCGAATCGCACCATCTGGATCAAACACGATGAGTTGATCATTGGCAACCAGGCAAGCGAAGTTCG<br>CGCCGCGCCGATCTTCCCGGAATATACTGTCTCGTGGATCGAAAAAGAGATTGATGATCTGGCAGATC<br>GTCCCGGTGCTGGCTTTGCGGTGAGCGAAGAGAACAAACGCGTTCTGCATGAAGTGTGCCCGTGGTGG<br>CGCGGTCAGACCGTACAGGATCGCTGCTACGGCATGTTTACCGATGAGCAAAAAGGTCTGCTGGCGAC<br>CGGAATCATTAAAGCGGAAGGCAATATGACCTCCGGCGATGCGCACCTGGCGGTGAATTTCCCGCTGC<br>TGCTGGAAAAAGGGCTTGATGGTCTGCGCGAGGAAGTAGCGGAACGTCGCTCGCGCATCAACCTGAC<br>GGTGCTGGAAGATTTACACGGTGAGCAATTCCTGAAAGCGATTGATATCGTGCTGGTGGCAGTCAGTG<br>AACACATTGAACGTTTCGCTGCCCTGGCGCGTGAAATGGCCGCGACCGAAACCCGCGAAAGCCGTCGC<br>GATGAACTGCTGGCGATGGCAGAAAACTGCGATCTTATCGCCCACCAGCCGCCGCAGACTTTCTGGCA<br>GGCGCTGCAACTGTGTTACTTCATCCAGTTGATTTTGCAGATCGAATCTAACGGTCACTCAGTATCGTT<br>TGGTCGTATGGACCAGTATCTCTACCCGTACTATCGCCGCGACGTTGAACTCAACCAGACGCTGGATC<br>GCGAACACGCCATCGAGATGCTGCATAGCTGCTGGCTGAAACTGCTGGAAGTGAACAAGATCCGCTCC<br>GGCTCACACTCAAAAGCCTCTGCGGGAAGTCCGCTGTATCAGAACGTCACTATTGGCGGGCAAAATCT<br>GGTTGATGGTCAACCAATGGACGCGGTGAATCCACTCTCTTACGCGATCCTCGAATCCTGCGGTCGCC<br>TGCGTTCCACTCAGCCTAACCTCAGCGTGCGTTACCATGCAGGAATGAGCAACGATTTCCTCGACGCC<br>TGCGTACAGGTGATCCGTTGCGGCTTCGGGATGCCGGCGTTCAACAACGACGAAATCGTGATCCCGGA<br>ATTTATTAAACTCGGTATTGAACCGCAGGACGCTTATGACTACGCAGCGATTGGTTGTATAGAAACCG<br>CCGTCGGTGGCAAATGGGGCTATCGCTGTACCGGCATGAGCTTTATCAACTTCGCCCGCGTGATGCTG<br>GCGGCGCTGGAAGGCGGGCATGATGCCACCAGCGGCAAAGTGTTCCTGCCACAAGAAAAAGCGTTGT<br>CGGCAGGTAACTTCAACAACTTCGATGAAGTGATGGACGCGTGGGATACGCAAATCCGTTACTACACC<br>CGCAAATCAATCGAAATCGAATATGTCGTCGACACCATGCTGGAAGAGAACGTGCACGATATTCTCTG<br>CTCGGCGCTGGTGGATGACTGTATTGAGCGAGCGAAAAGTATCAAGCAAGGCGGCGCGAAATATGAC<br>TGGGTTTCTGGCCTGCAGGTCGGCATTGCCAACCTCGGCAACAGCCTGGCGGCAGTGAAGAAACTGGT<br>GTTTGAACAAGGTGCGATTGGTCAGCAACAGCTTGCTGCCGCACTGGCAGATGACTTCGACGGCCTGA<br>CTCACGAGCAGCTGCGTCAGCGGCTGATTAACGGTGCGCCGAAGTACGGCAACGACGATGATACTGTC

	GATACGCTGCTGGCTCGCGCTTATCAGACCTATATCGACGAACTGAAACAGTACCATAATCCGCGCTA<br>CGGTCGTGGTCCGGTTGGCGGCAACTATTACGCGGGTACGTCATCAATCTCCGCTAACGTACCGTTTG<br>GCGCGCAGACTATGGCAACACCGGACGGGCGTAAAGCCCACACCCCGCTGGCAGAAGGCGCAAGCCC<br>GGCCTCCGGTACTGACCATCTTGGCCCTACTGCGGTCATTGGCTCAGTGGGTAAACTGCCTACGGCAG<br>CGATTCTCGGCGGCGTGTTGCTCAACCAGAAACTGAATCCGGCAACGCTGGAGAACGAATCTGACAA<br>GCAGAAACTGATGATCCTGCTGCGTACCTTCTTTGAAGTGCATAAAGGCTGGCATATTCAGTACAACA<br>TCGTTTCCCGCGAAACGCTGCTGGATGCGAAAAAACATCCCGATCAGTATCGCGATCTGGTAGTGCGT<br>GTCGCGGGCTATTCCGCGTTCTTCACCGCGCTCTCTCCAGACGCTCAGGACGATATCATCGCCCGTACT<br>GAACATATGCTGTAAACTAGTCC
pflB	GGAATTCCATATGCATCACCATCATCACCACATGTCCGAGCTTAATGAAAAGTTAGCCACAGCCTGGG<br>AAGGTTTTACCAAAGGTGACTGGCAGAATGAAGTAAACGTCCGTGACTTCATTCAGAAAAACTACACT<br>CCGTACGAGGGTGACGAGTCCTTCCTGGCTGGCGCTACTGAAGCGACCACCACCCTGTGGGACAAAGT<br>AATGGAAGGCGTTAAACTGGAAAACCGCACTCACGCGCCAGTTGACTTTGACACCGCTGTTGCTTCCA<br>CCATCACCTCTCACGACGCTGGCTACATCAACAAGCAGCTTGAGAAAATCGTTGGTCTGCAGACTGAA<br>GCTCCGCTGAAACGTGCTCTTATCCCGTTCGGTGGTATCAAAATGATCGAAGGTTCCTGCAAAGCGTA<br>CAACCGCGAACTGGATCCGATGATCAAAAAAATCTTCACTGAATACCGTAAAACTCACAACCAGGGC<br>GTGTTCGACGTTTACACTCCGGACATCCTGCGTTGCCGTAAATCTGGTGTTCTGACCGGTCTGCCAGAT<br>GCATATGGCCGTGGCCGTATCATCGGTGACTACCGTCGCGTTGCGCTGTACGGTATCGACTACCTGAT<br>GAAAGACAAACTGGCACAGTTCACTTCTCTGCAGGCTGATCTGGAAAACGGCGTAAACCTGGAACAG<br>ACTATCCGTCTGCGCGAAGAAATCGCTGAACAGCACCGCGCTCTGGGTCAGATGAAAGAAATGGCTG<br>CGAAATACGGCTACGACATCTCTGGTCCGGCTACCAACGCTCAGGAAGCTATCCAGTGGACTTACTTC<br>GGCTACCTGGCTGCTGTTAAGTCTCAGAACGGTGCTGCAATGTCCTTCGGTCGTACCTCCACCTTCCTG<br>GATGTGTACATCGAACGTGACCTGAAAGCTGGCAAGATCACCGAACAAGAAGCGCAGGAAATGGTTG<br>ACCACCTGGTCATGAAACTGCGTATGGTTCGCTTCCTGCGTACTCCGGAATACGATGAACTGTTCTCTG<br>GCGACCCGATCTGGGCAACCGAATCTATCGGTGGTATGGGCCTCGACGGTCGTACCCTGGTTACCAAA<br>AACAGCTTCCGTTTCCTGAACACCCTGTACACCATGGGTCCGTCTCCGGAACCGAACATGACCATTCTG<br>TGGTCTGAAAAACTGCCGCTGAACTTCAAGAAATTCGCCGCTAAAGTGTCCATCGACACCTCTTCTCTG<br>CAGTATGAGAACGATGACCTGATGCGTCCGGACTTCAACAACGATGACTACGCTATTGCTTGCTGCGT

	AAGCCCGATGATCGTTGGTAAACAAATGCAGTTCTTCGGTGCGCGTGCAAACCTGGCGAAAACCATGCTGTACGCAATCAACGGCGGCGTTGACGAAAAACTGAAAATGCAGGTTGGTCCGAAGTCTGAACCGATCAAAGGCGATGTCCTGAACTATGATGAAGTGATGGAGCGCATGGATCACTTCATGGACTGGCTGGCTAAACAGTACATCACTGCACTGAACATCATCCACTACATGCACGACAAGTACAGCTACGAAGCCTCTCTGATGGCGCTGCACGACCGTGACGTTATCCGCACCATGGCGTGTGGTATCGCTGGTCTGTCCGTTGCTGCTGACTCCCTGTCTGCAATCAAATATGCGAAAGTTAAACCGATTCGTGACGAAGACGGTCTGGCTATCGACTTCGAAATCGAAGGCGAATACCCGCAGTTTGGTAACAATGATCCGCGTGTAGATGACCTGGCTGTTGACCTGGTAGAACGTTTCATGAAGAAAATTCAGAAACTGCACACCTACCGTGACGCTATCCCGACTCAGTCTGTTCTGACCATCACTTCTAACGTTGTGTATGGTAAGAAAACGGGTAACACCCCAGACGGTCGTCGTGCTGGCGCGCCGTTCGGACCGGGTGCTAACCCGATGCACGGTCGTGACCAGAAAGGTGCAGTAGCCTCTCTGACTTCCGTTGCTAAACTGCCGTTTGCTTACGCTAAAGATGGTATCTCCTACACCTTCTCTATCGTTCCGAACGCACTGGGTAAAGACGACGAAGTTCGTAAGACCAACCTGGCTGGTCTGATGGATGGTTACTTCCACCACGAAGCATCCATCGAAGGTGGTCAGCACCTGAACGTTAACGTGATGAACCGTGAAATGCTGCTCGACGCGATGGAAAACCCGGAAAAATATCCGCAGCTGACCATCCGTGTATCTGGCTACGCAGTACGTTTCAACTCGCTGACTAAAGAACAGCAGCAGGACGTTATTACTCGTACCTTCACTCAATCTATGTAAACTAGTCC
pflD	GGAATTCCATATGCATCACCATCATCACCACACGAATCGTATCTCTCGCCTCAAAACTGCACTGTTTGCCAATACCCGTGAAATCTCGCTGGAGCGGGCGCTGCTTTATACCGCCAGCCATCGGCAAACCGAAGGCGAACCGGTGATATTGCGCCGGGCGAAAGCAACAGCGTATATCCTTGAACATGTTGAAATTTCGATTCGTGATGAAGAACTGATTGCCGGTAACCGCACCGTAAAACCGCGCGCCGGGATTATGTCGCCGGAAATGGACCCTTACTGGCTGCTGAAAGAGCTGGATCAATTCCCGACGCGTCCGCAGGACCGCTTTGCTATCAGCGAAGAAGATAAACGTATCTACCGTGAAGAGTTGTTCCCGTACTGGGAAAAACGTTCGATGAAAGATTTCATCAACGGGCAGATGACAGATGAAGTAAAAGCCGCGACCAACACGCAGATTTTCAGCATCAACCAGACGGATAAAGGCCAGGGGCACATTATTATTGATTACCCACGCCTGTTGAATCACGGGCTGGGTGAGCTGGTGGCACAGATGCAGCAACATTGTCAGCAACAGCCGGAGAATCACTTTTATCAGGCCGCGTTGTTACTGCTGGAAGCCTCGCAGAAACACATTTTGCGTTACGCCGAACTGGCGGAAACGATGGCGGCAAACTGCACAGATGCCCAGCGTCGCGAAGAGCTGCTGACTATTGCAGAGATCTCCCGCCATAACGCGCAACATAAGCCGCAGACGTTCTGGCAGGCGTGCCAGTTATTCTGGTACATGAACATCATTCTGCAATACGAATCC

	AACGCCAGTTCGCTATCGTTGGGGCGCTTCGACCAGTATATGTTGCCGTTCTATCAGACATCATTAACC
	CAGGGCGAAGATGCGGCGTTCCTGAAAGAACTGCTCGAATCTTTATGGGTGAAATGCAACGACATCGT
	GCTGTTGCGCTCCACCAGTAGCGCGCGTTATTTCGCCGGTTTCCCGACCGGCTATACCGCACTGCTCGG
	CGGGTTAACCGAGAACGGACGTAGCGCGGTGAACGTGCTTTCGTTCCTTTGCCTTGACGCCTATCAAA
	GCGTGCAATTACCGCAACCGAACCTCGGCGTGCGCACTAACGCCTTGATCGACACGCCGTTCCTGATG
	AAAACCGCCGAAACCATTCGCTTCGGTACCGGTATTCCGCAAATCTTTAACGATGAAGTGGTGGTGCC
	AGCGTTCCTCAACCGTGGCGTTTCGCTGGAAGATGCGCGCGACTATTCCGTAGTGGGCTGTGTGGAAT
	TATCTATTCCCGGCAGAACCTACGGCTTGCATGACATCGCGATGTTTAATCTGCTGAAAGTGATGGAA
	ATCTGCCTGCATGAAAATGAAGGCAATGCTGCGCTGACTTATGAAGGTTTACTGGAGCAGATCCGCGC
	CAAGATCAGCCACTACATCACCCTGATGGTTGAGGGCAGCAATATTTGTGATATCGGCCATCGCGACT
	GGGCACCTGTACCGCTGCTCTCATCGTTTATCAGCGATTGTCTGGAAAAAGGCCGCGATATTACCGAT
	GGCGGCGCGCGTTATAACTTCTCCGGCGTACAGGGGATCGGTATCGCCAACCTGAGCGATTCTCTCCA
	TGCGTTGAAAGGGATGGTTTTTGAGCAACAGCGTTTAAGTTTTGACGAATTGCTGTCGGTATTAAAAG
	CCAACTTCGCAACGCCAGAAGGCGAAAAAGTCCGCGCTCGCTTAATTAACCGCTTCGAGAAATACGGT
	AACGATATCGACGAGGTGGATAACATCAGCGCCGAACTGTTGCGCCACTACTGCAAAGAAGTGGAAA
	AATACCAGAACCCGCGCGGCGGCTACTTCACGCCGGGATCGTATACCGTTTCTGCTCACGTCCCGTTG
	GGATCGGTGGTTGGCGCGACGCCAGACGGTCGTTTTGCCGGAGAACAGCTGGCAGACGGCGGCTTGTC
	ACCCATGCTGGGCCAGGACGCACAAGGGCCAACAGCGGTACTGAAGTCAGTCAGTAAGCTCGATAAC
	ACGCTGCTGTCTAACGGTACGTTGCTGAACGTGAAATTCACTCCGGCGACCCTGGAAGGTGAAGCAGG
	ATTACGCAAACTGGCCGACTTCTTACGGGCGTTTACCCAGCTTAAGTTACAACATATTCAGTTTAACGT
	GGTGAACGCCGACACGTTGCGGGAAGCGCAACAGCGCCCACAAGATTATGCCGGGCTGGTGGTGCGC
	GTTGCCGGATACAGCGCCTTCTTTGTCGAACTGTCGAAGGAGATCCAGGATGACATCATCCGCCGGAC
	AGCGCATCAGCTGTAAACTAGTCC
tdcE	GGAATTCCATATGCATCACCATCATCACCACAAGGTAGATATTGATACCAGCGATAAGCTGTACGCCG
	ACGCATGGCTTGGCTTTAAAGGTACGGACTGGAAAAACGAAATTAATGTCCGCGATTTTATTCAACAT
	AACTATACACCGTATGAAGGCGATGAATCTTTCCTCGCCGAAGCGACGCCTGCCACCACGGAATTGTG
	GGAAAAAGTAATGGAAGGCATCCGTATCGAAAATGCAACCCACGCGCCGGTTGATTTCGATACCAAT
	ATTGCCACCACAATTACCGCTCATGATGCGGGATATATTAACCAGCCGCTGGAAAAAATTGTTGGCCT

GCAAACGGATGCGCCGTTGAAACGTGCGCTACACCCGTTCGGTGGCATTAATATGATTAAAAGTTCAT

TCCACGCCTATGGCCGAGAAATGGACAGTGAATTTGAATATCTGTTTACCGATCTGCGTAAAACCCAT

AACCAGGGCGTATTTGATGTTTACTCACCGGATATGCTGCGCTGCCGTAAATCTGGCGTGCTGACCGG

TTTACCAGATGGCTATGGCCGTGGGCGCATTATCGGTGACTATCGCCGCGTAGCGCTGTATGGCATCA

GTTATCTGGTACGTGAACGCGAACTGCAATTTGCCGATCTCCAGTCTCGTCTGGAAAAAGGCGAGGAT

CTGGAAGCCACCATCCGTCTGCGTGAGGAGCTGGCAGAGCATCGTCATGCGCTGTTGCAGATTCAGGA

AATGGCGGCGAAATATGGCTTTGATATCTCTCGCCCGGCGCAGAATGCGCAGGAAGCGGTGCAGTGG

CTCTACTTCGCTTATCTGGCGGCAGTGAAATCGCAAAATGGCGGCGCGATGTCGCTGGGCCGCACGGC

ATCGTTCCTCGATATCTACATTGAGCGCGACTTTAAAGCTGGCGTACTCAATGAGCAGCAGGCACAGG

AACTGATCGATCACTTCATCATGAAGATCCGTATGGTACGCTTCCTGCGTACACCGGAATTTGATTCGC

TGTTCTCCGGCGACCCAATCTGGGCGACGGAAGTGATCGGCGGGATGGGGCTGGACGGTCGTACGCTG

GTGACCAAAAACTCCTTCCGCTATTTGCACACCCTGCACACTATGGGGCCGGCACCGGAACCTAACCT

GACCATTCTTTGGTCGGAAGAATTACCGATTGCCTTCAAAAAATATGCCGCGCAGGTGTCGATCGTCA

CCTCTTCCTTGCAGTATGAAAATGACGATCTGATGCGTACTGACTTCAACAGCGACGATTACGCGATT

GCCTGCTGCGTCAGCCCAATGGTGATTGGTAAGCAAATGCAGTTCTTTGGTGCACGCGCTAACCTGGC

GAAAACGCTGCTCTACGCAATTAACGGCGGGGTGGACGAGAAGCTGAAGATTCAGGTCGGGCCGAAA

ACAGCACCGCTGATGGACGACGTGCTGGATTACGACAAAGTGATGGACAGCCTCGATCACTTCATGGA

CTGGCTGGCGGTGCAGTACATCAGCGCGCTGAATATCATTCACTACATGCACGACAAGTACAGCTACG

AAGCTTCGCTGATGGCGCTGCACGATCGTGATGTCTATCGCACTATGGCATGCGGCATCGCGGGCCTG

TCGGTGGCGACGGACTCCCTGTCTGCCATCAAATATGCCCGCGTGAAACCAATCCGTGACGAAAACGG

CCTGGCGGTGGACTTTGAAATCGACGGTGAATATCCGCAGTACGGCAACAACGACGAGCGCGTAGAC

AGCATTGCCTGCGACCTGGTTGAACGCTTTATGAAGAAAATTAAAGCGCTGCCAACCTATCGCAACGC

CGTCCCTACCCAGTCGATTCTGACTATCACTTCTAACGTGGTGTACGGCCAGAAAACCGGTAATACGC

CGGACGGTCGTCGCGCCGGAACACCGTTCGCGCCGGGCGCTAACCCGATGCATGGTCGTGACCGCAA

AGGTGCCGTGGCCTCATTGACGTCGGTGGCGAAACTGCCGTTCACCTACGCCAAAGATGGGATCTCGT

ACACCTTCTCAATCGTTCCTGCGGCGCTGGGCAAAGAAGATCCAGTACGTAAAACCAACCTTGTCGGC

CTGCTGGATGGGTATTTCCACCACGAAGCGGATGTCGAAGGCGGTCAACACCTCAACGTCAACGTAAT

GAATCGGGAAATGCTGCTGGATGCCATCGAGCACCCGGAAAAATATCCTAACCTGACAATCCGTGTCT

	CTGGCTACGCCGTGCGCTTCAACGCACTGACCCGTGAACAGCAACAGGATGTTATTTCACGTACCTTT ACCCAGGCGCTCTGAACTAGTCC

Table S3. GC analyses of substrates and products.

Substrate	Retention time [min]	Product	Retention time [min]
2-pentanone	4.5	pentan-2-amine	4.3
4-phenyl-2-butanone	16.1	4-phenylbutan-2-amine	21.6
cyclohexanone	7.1	cyclohexylamine	8.5

Table S4. Chiral analyses of the products.

Product	Retention time [min] (*S*)	Retention time [min] (*R*)
Pentan-2-amine	9.4	9.6
4-phenylbutan-2-amine	34.5	34.9

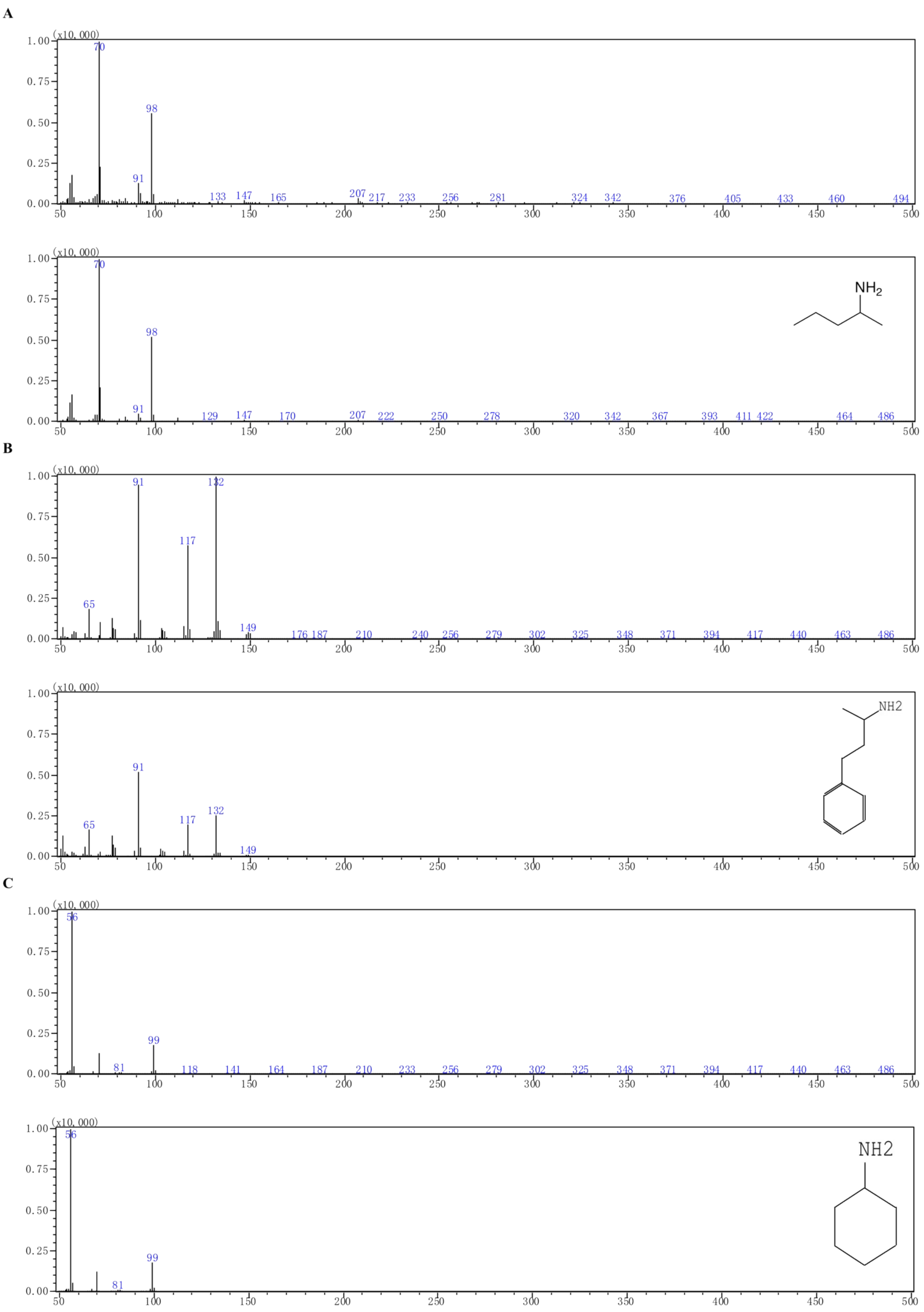

Figure S1. Mass spectrometry analyses.

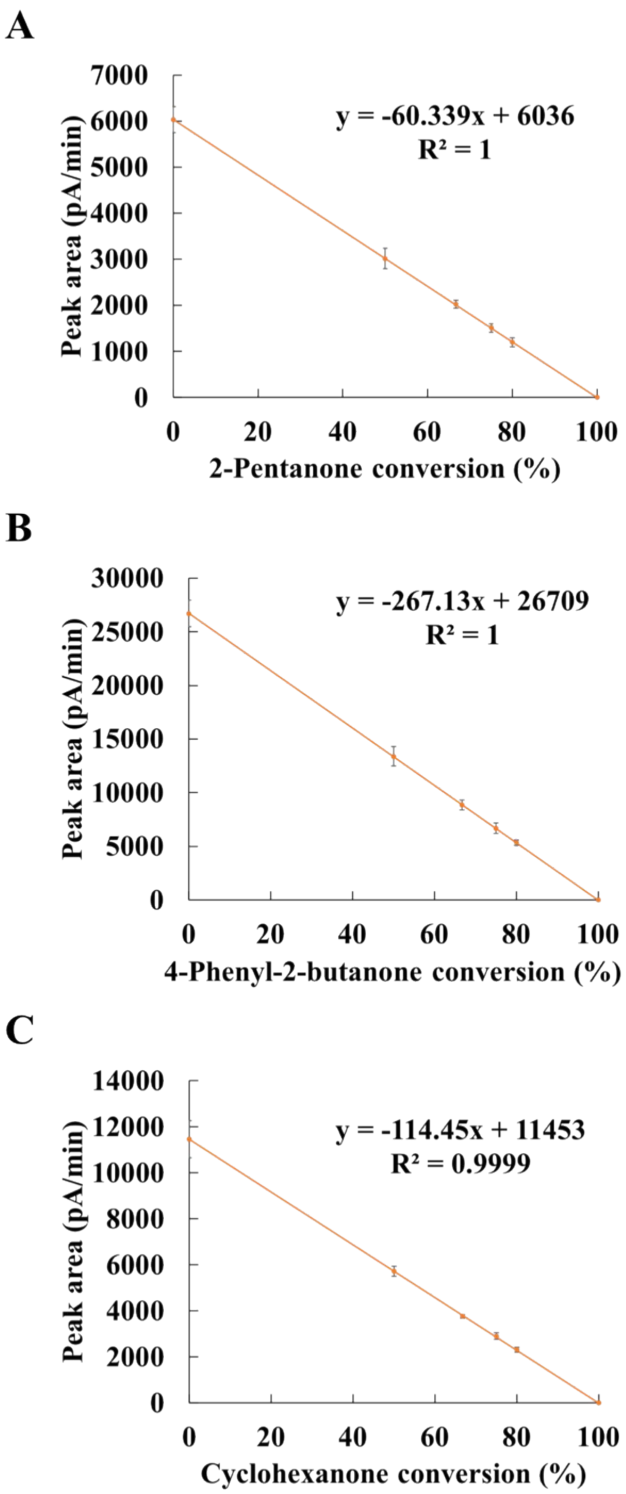

Figure S2. The standard curves of substrates. (**A-C**) The standard curves of 2-pentanone, 4-phenyl-2-butanone and cyclohexanone, respectively. Results are represented as mean ± SD of three replicates.

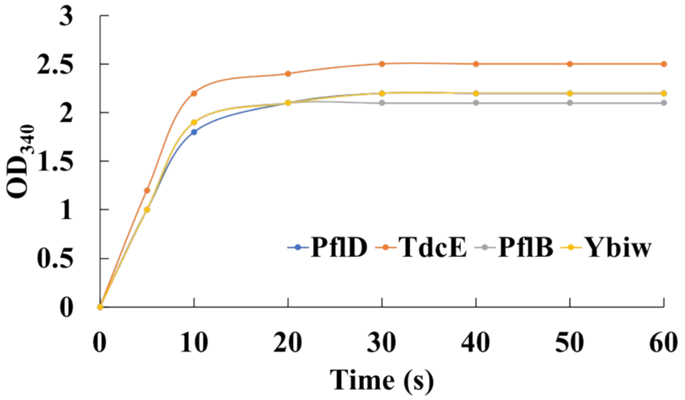

Figure S3. The profiles of OD_{340} versus time.

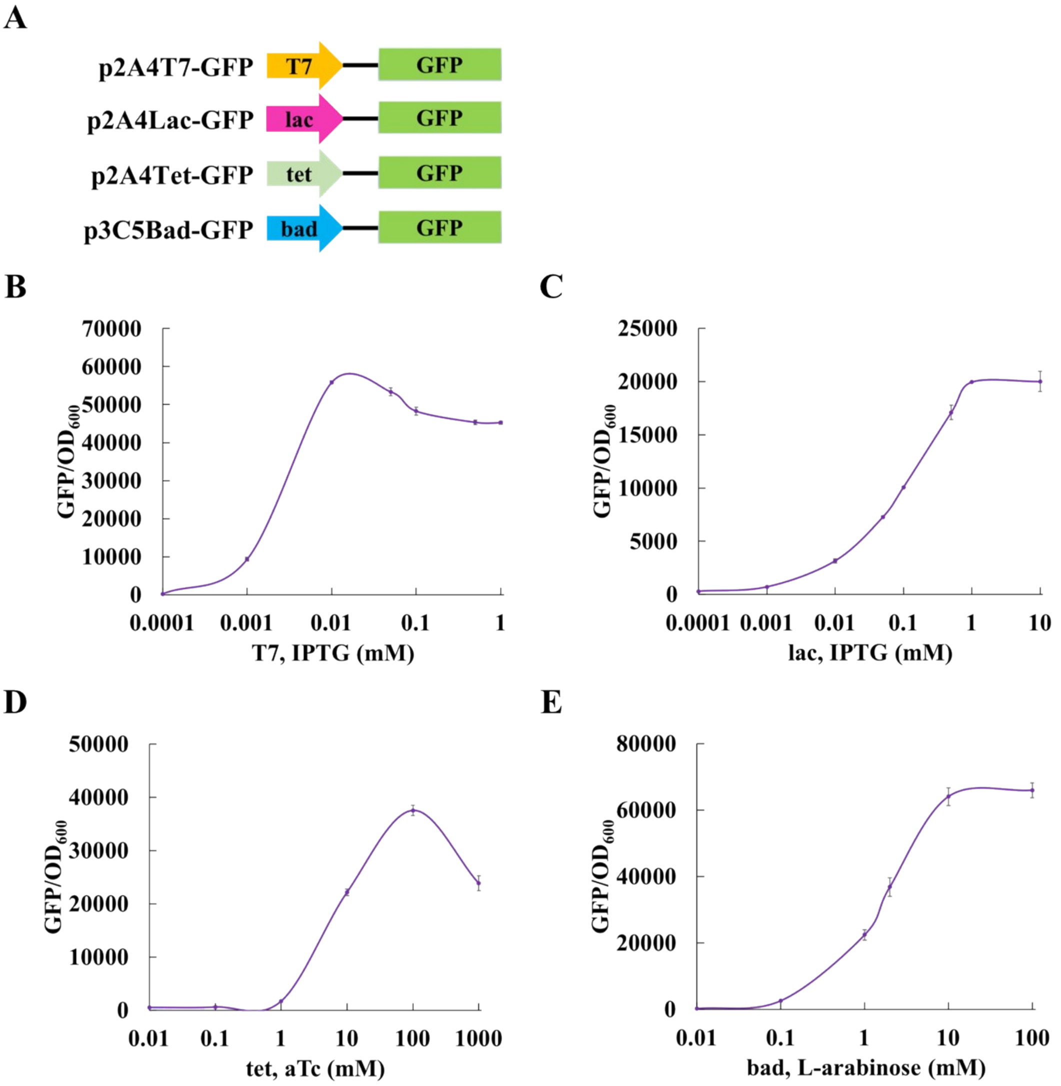

Figure S4. Expression of GFP with different promoters in *E. coli*. (**A**) Schematic diagram of plasmids used for GFP expression. (**B-E**) GFP/OD_{600} results from cultures of *E. coli* containing indicated promoters with inducing the expression of GFP at 25℃ for 16 h. Results are represented by the mean values of three replicates and the error bar by the standard deviation.
